# Supplementary material for: Transcriptome Analysis of Flower Development and Mining of Genes Related to Flowering Time in Tomato (Solanum lycopersicum)
Source: Int J Mol Sci. 2021 Jul 29;22(15):8128. doi: 10.3390/ijms22158128 (PMC8347202; doi:10.3390/ijms22158128)
Supplement: Supplementary file 1 [file ijms-22-08128-s001.zip › Figure S1.pdf]

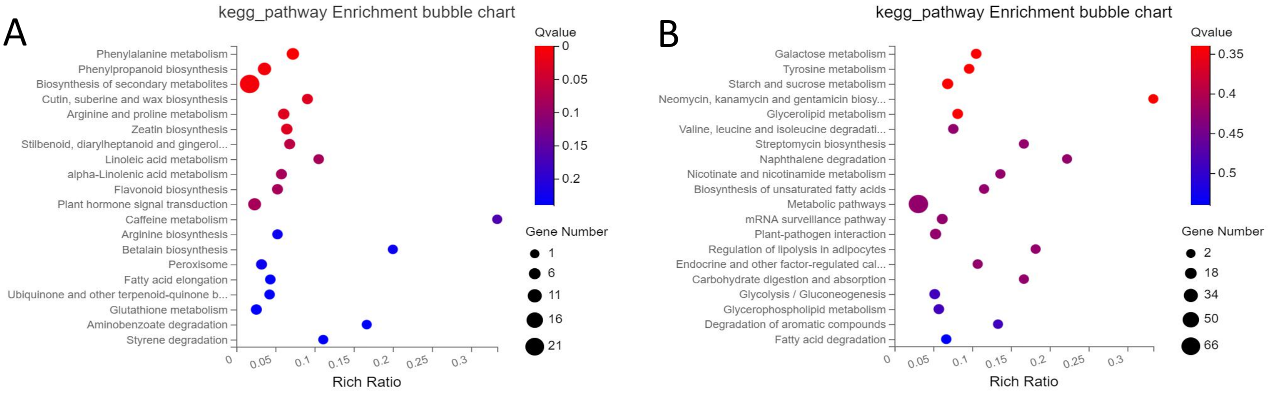

Figure S1. KEGG enrichment map of four modules from WGCNA. (A) KEGG pathways enriched by the MEgreenyellow. (B) KEGG pathways enriched by the MEorange. The rich factor is the ratio of differentially expressed gene numbers annotated in this pathway term to all gene numbers annotated in this pathway term. The greater the Rich factor, the greater the degree of pathway enrichment.
